# Current understanding of the interplays between host hormones and plant viral infections

**DOI:** 10.1371/journal.ppat.1009242

**Published:** 2021-02-25

**Authors:** Shanshan Zhao, Yi Li

**Affiliations:** 1 State Key Laboratory of Protein and Plant Gene Research, School of Life Sciences, Peking University, Beijing, China; 2 Vector-borne Virus Research Center, State Key Laboratory of Ecological Pest Control for Fujian and Taiwan Crops, Fujian Province Key Laboratory of Plant Virology, Institute of Plant Virology, Fujian Agriculture and Forestry University, Fuzhou, China; Virginia Polytechnic Institute and State University, UNITED STATES

## Abstract

Phytohormones mediate plant development and responses to stresses caused by biotic agents or abiotic factors. The functions of phytohormones in responses to viral infection have been intensively studied, and the emerging picture of complex mechanisms provides insights into the roles that phytohormones play in defense regulation as a whole. These hormone signaling pathways are not simple linear or isolated cascades, but exhibit crosstalk with each other. Here, we summarized the current understanding of recent advances for the classical defense hormones salicylic acid (SA), jasmonic acid (JA), and ethylene (ET) and also the roles of abscisic acid (ABA), auxin, gibberellic acid (GA), cytokinins (CKs), and brassinosteroids (BRs) in modulating plant–virus interactions.

## Introduction

During their growth and development, plants are frequently challenged by pathogens such as viruses, bacteria, fungi, oomycetes, nematodes, and insects [1]. Most plants are unable to avoid these negative influences by changing their spatial location, and they contain no specialized and mobile immune response cells with which to eliminate invasive diseases. For their survival and reproduction, plants have developed various highly efficient defense systems in the long and frequent battle against viruses [1–3].

**Fig 1:**
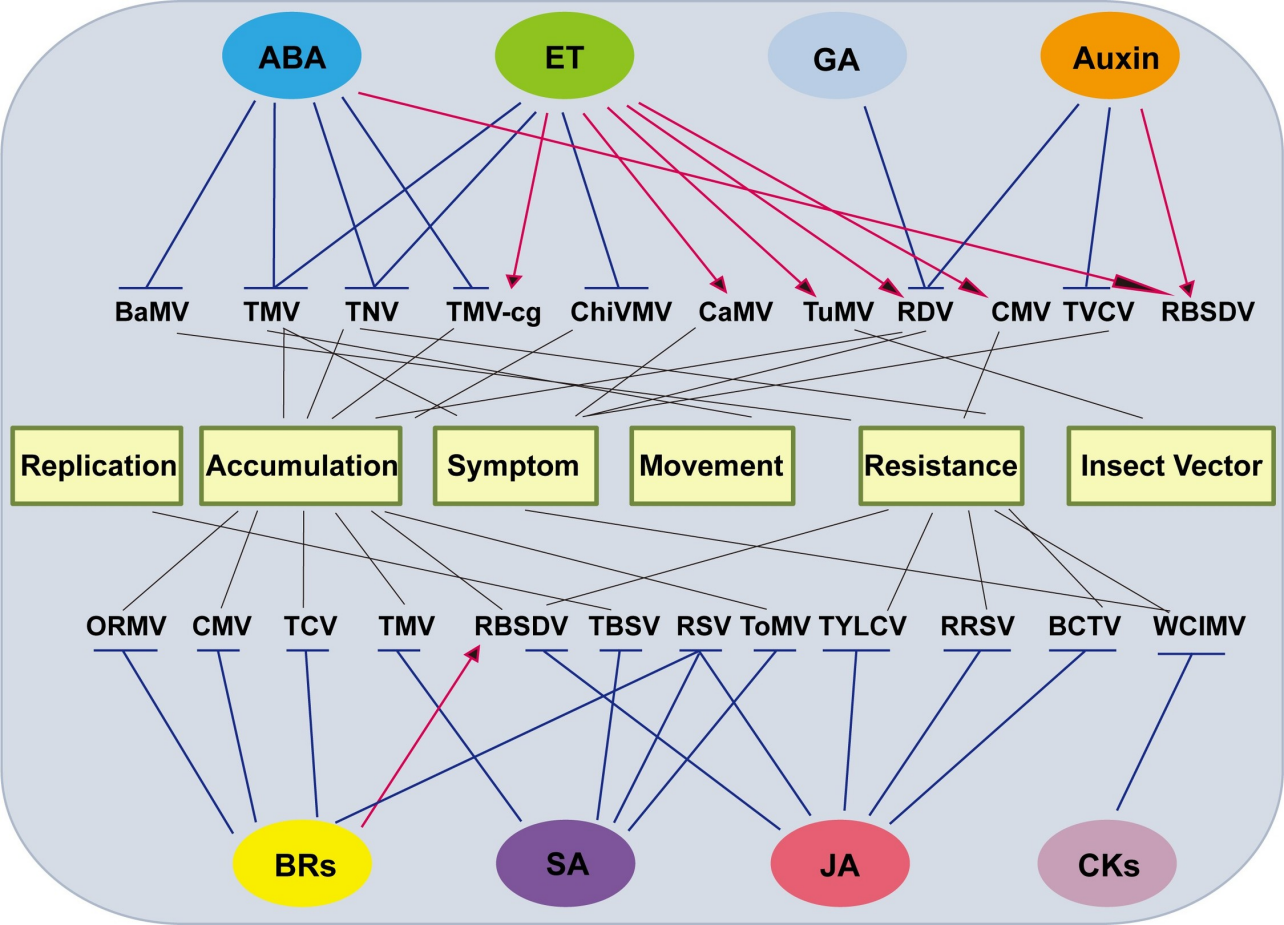
The roles of plant hormones in virus infections. The plant hormones shown in ovals generally have positive (red arrows) or negative (blue blocked lines) effects on different viruses in terms of replication, accumulation, symptom development, virus movement, host resistance, and the relationship between virus and insect vectors. For details, see text. ABA, abscisic acid; BaMV, bamboo mosaic virus; BCTV, beet curly top virus; BR, brassinosteroid; CaMV, cauliflower mosaic virus; ChiVMV, Chilli veinal mottle virus; CK, cytokinin; CMV, cucumber mosaic virus; ET, ethylene; GA, gibberellic acid; JA, jasmonic acid; ORMV, oilseed rape mosaic virus; RBSDV, rice black streaked dwarf virus; RDV, rice dwarf virus; RRSV, rice ragged stunt virus; RSV, rice stripe virus; SA, salicylic acid; TBSV, tomato bushy stunt virus; TCV, turnip crinkle virus; TMV, tobacco mosaic virus; TNV, tobacco necrosis virus; ToMV, tomato mosaic virus; TuMV, turnip mosaic virus; TVCV, turnip vein clearing virus; TYLCV, tomato yellow leaf curl virus; WClMV, white clover mosaic virus.

Despite having simple molecular structures and low cellular concentrations, phytohormones regulate most plant physiological processes, such as cell growth and division, plant growth and morphogenesis, as well as organogenesis and apoptosis [4]. Phytohormones are also involved in plant defense against viruses [3,5] (Fig 1), and the roles of salicylic acid (SA), jasmonic acid (JA), ethylene (ET) in plant–virus interactions have been extensively reported [6–10]. In addition, functions of abscisic acid (ABA), auxins, gibberellic acid (GA), cytokinins (CKs) and brassinosteroids (BRs) in plant defense are also gradually being elucidated [11–17].

**Fig 2:**
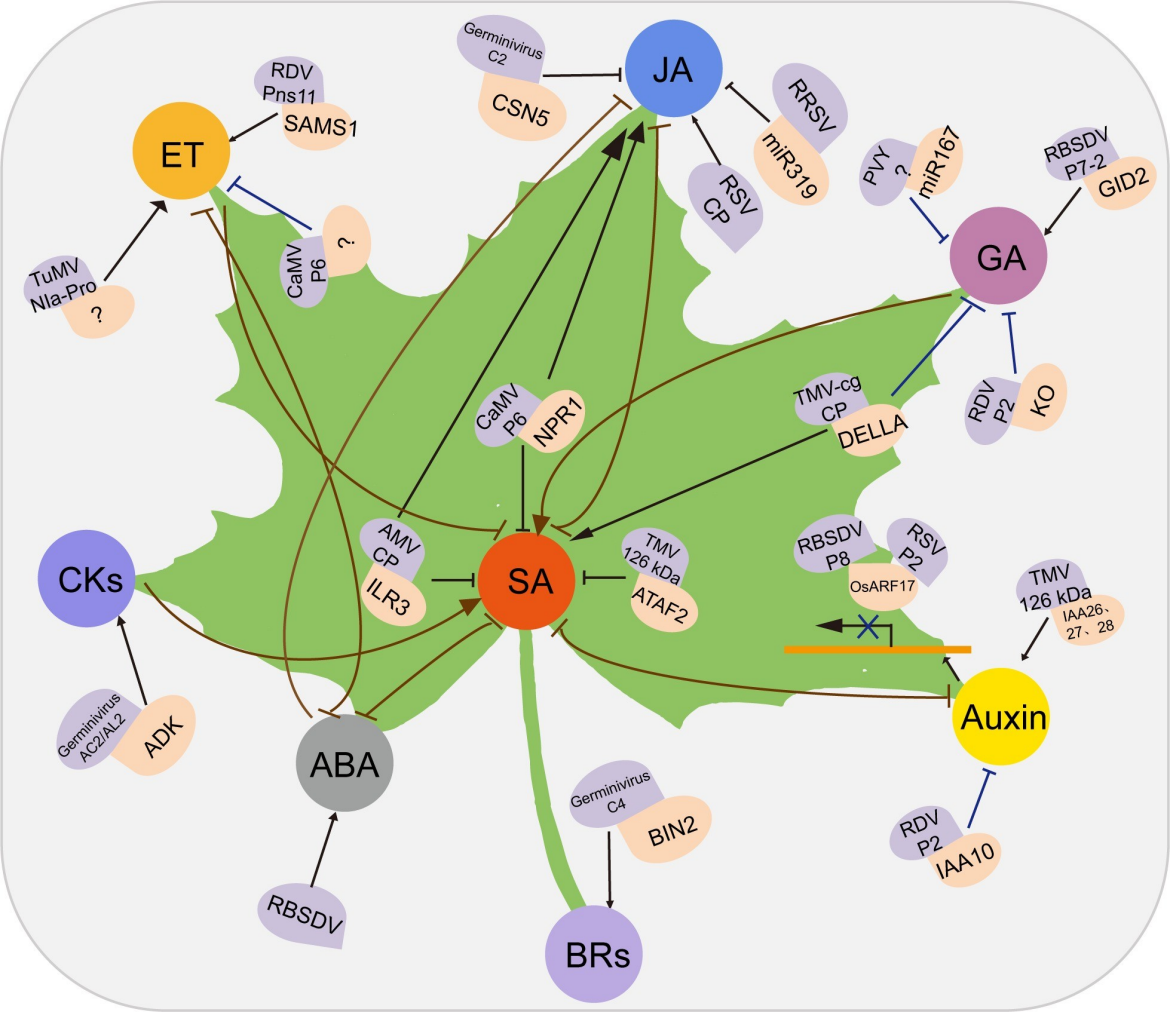
Plant hormone–virus interactions. Different virus proteins and host factors interact and lead to positive (arrows) or negative (blocked lines) effects on plant hormone biosynthesis or signaling pathways. The roles of SA, JA, and ET in plant defense responses have been intensively studied. Auxin and GAs are mainly related to the host phenotype after virus infection. CKs and BRs are involved in plant–virus interactions. Different plant hormones display synergetic or antagonistic crosstalk during plant–virus interactions, and microRNAs are used by viruses to target plant hormone pathways. For details, see text. ABA, abscisic acid; AMV, alfalfa mosaic virus; BIN2, BRASSINOSTEROID-INSENSITIVE 2; BR, brassinosteroid; CaMV, cauliflower mosaic virus; CK, cytokinin; ET, ethylene; GA, gibberellic acid; GID2, GIBBERELLIN-INSENSITIVE DWARF2; JA, jasmonic acid; PVY, potato virus Y; RBSDV, rice black streaked dwarf virus; RDV, rice dwarf virus; RRSV, rice ragged stunt virus; RSV, rice stripe virus; SA, salicylic acid; SAMS1, S-adenosyl-l-methionine synthase 1; TMV, tobacco mosaic virus; TuMV, turnip mosaic virus.

Viral infection can disrupt hormonal pathways, which manifests as the simultaneous induction of synergistic or antagonistic hormones and the triggering of defense responses [10,13,15,16,18] (Fig 2). Such alterations often lead to the appearance of symptoms and are closely related to viral movement, replication, and systemic infection. Thus, a comprehensive understanding of hormone-related functions in plant–virus pathosystems may facilitate innovative biotechnological, genetic, and breeding approaches for crop protection and improvement [3,5]. Here, we discuss the regulatory mechanisms involving different phytohormones in plant–virus interactions. We also focus on the crosstalk among the different hormone signaling systems that fine-tune defense responses.

### SA/JA and plant–virus interactions

The ability of plants to successfully resist pathogen invasion relies on crosstalk among different hormone signaling pathways [3,5,19]. Notably, crosstalk between SA and JA participates in plant defense responses, although the contribution of SA/JA signaling molecules in plant defense differs and depends on the type of invading pathogen [3,5,7]. SA-dependent defense is generally effective against biotrophic pathogens, whereas JA-dependent defense is usually triggered in response to necrotrophic pathogens [5]. These 2 signaling pathways influence each other via a complex network of synergistic and antagonistic interactions, which have been extensively studied and recently reviewed [3,5,7].

### SA-mediated effects

SA is a phenolic compound that is widely present in plants and regulates numerous physiological processes and adaptive stress responses [20,21]. It was identified to be associated with plant defense over 30 years ago, based on its effects on virus infection. Since then, much attention has been focused on its role in plant–virus interactions [21]. Depending upon the virus–plant combination, SA can affect 3 main stages of the virus infection cycle within the infected plants: intercellular trafficking, long-distance movement, and replication [3,5,21]. For example, SA inhibits the replication of tomato bushy stunt virus (TBSV) via a competitive interaction with cytosolic glycerol 3-phosphate dehydrogenase (GAPDH) [22]. Many studies on SA-mediated plant defense have demonstrated that SA is necessary not only to induce a hypersensitive response (HR), but also for the establishment of both local and systemic-acquired resistance (SAR). The induction of SA causes an increase in the expression of pathogenesis-related (PR) genes and leads to the accumulation of reactive oxygen species (ROS) and callose deposition during viral infection [5,23]. The SA-mediated repression of viral infection is also related to the RNA interference (RNAi) pathway: Campos and colleagues observed that expression of the *Arabidopsis* orthologs *SIDCL1*, *SIDCL2*, *SIRDR1*, and *SIRDR2* in tomato were significantly induced after SA treatment, resulting in a delay in the accumulation of tomato mosaic virus (ToMV) RNA in inoculated plants [24]. This finding indicates that SA can prime defense by pre-inducing genes related to the RNAi pathway. In a recent work, 1 *R* gene *STV11* encoding a sulfotransferase was identified through a forward genetic screen. STV11 can catalyze the conversion of SA to sulphonated SA (SSA), which is a more effective molecule in rice than SA to trigger resistance against rice stripe virus (RSV) and inhibit RSV replication [25]. How does SA treatment induce the expressions of *SIDCL1*, *SIDCL2*, *SIRDR1*, and *SIRDR2* in tomato and SSA inhibit RSV replication need further study.

### JA-mediated effects

**Fig 3:**
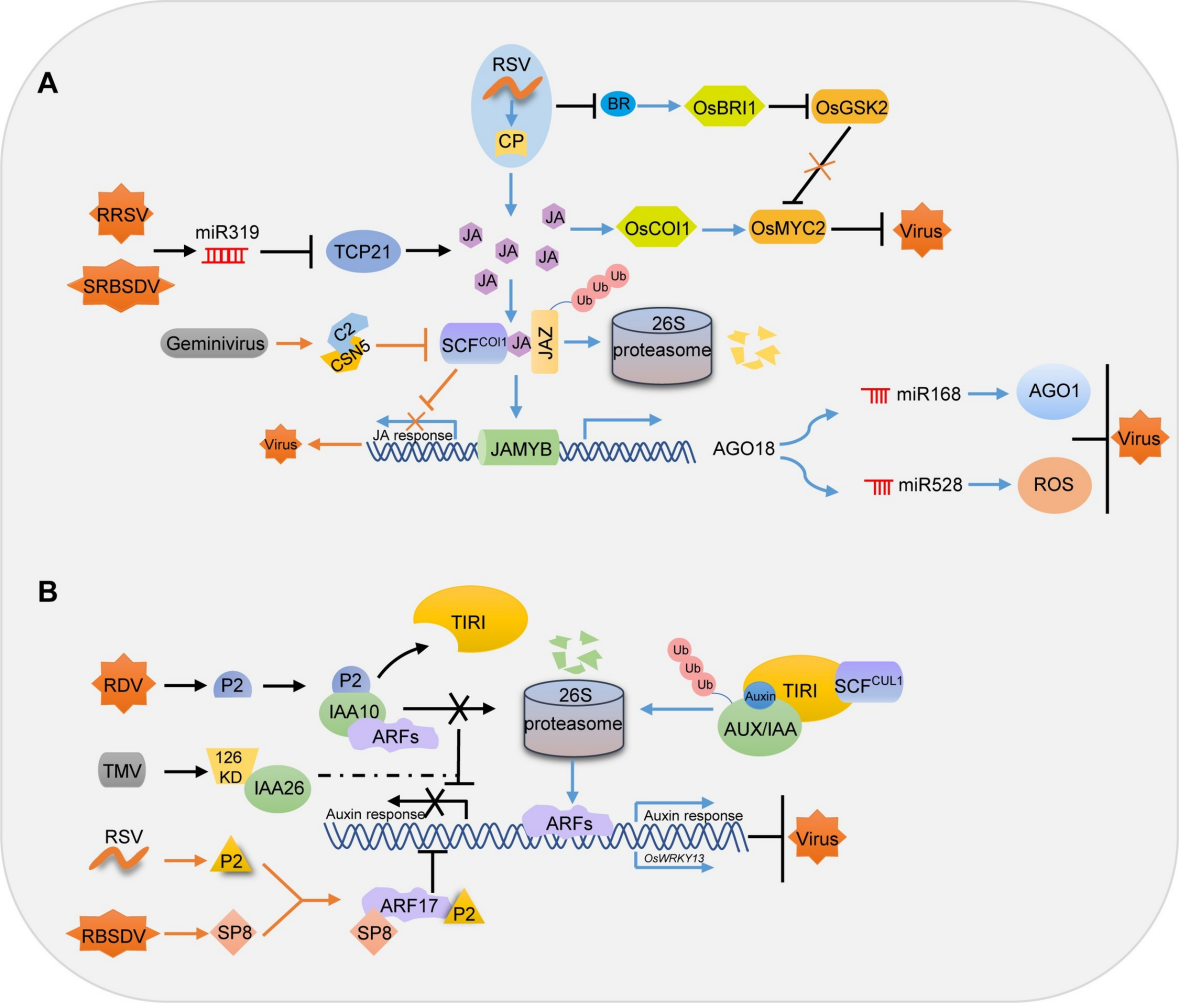
The role of JA and auxin in plant–virus interactions. (A) The role of JA in plant–virus interactions. Geminivirus C2 binds to CSN5 and affects the activity of SCFs, thus suppressing the JA response to viral infection [28]; RRSV and SRBSDV infection induces miR319, which reduces the function of *TCP21* and consequently suppressing JA-mediated defense to facilitate virus infection and symptom development [31]; The RSV CP increases the JA level, resulting in the degradation of JAZ proteins and the release of JAMYB. JAMYB binds to and activates the *AGO18* promoter, enhancing host antiviral defense by sequestering miR168 and miR528 and releasing AGO1 and ROS [27]. RSV infection also inhibits the BR signaling pathway and increases the accumulation of OsGSK2, which interacts with and phosphorylates OsMYC2 for degradation and therefore reduces JA-mediated RSV resistance [108]. (B) The role of auxin in plant–virus interactions. The TMV 126 kDa protein interacts with IAA26 and disrupts its localization, thus regulating auxin response during disease development [78]; The RDV P2 protein disrupts auxin signaling by interacting with OsIAA10, thus causing infected plants to display typical viral symptoms [14]; RSV P2 and RBSDV SP8 target OsARF17 and disrupt its dimerization and DNA binding activity, thus manipulating auxin signaling to facilitate infection [83]. AGO18, ARGONAUTE 18; BR, brassinosteroid; JA, jasmonic acid; JAMYB, JA-induced MYB; JAZ, JA ZIM domain; RBSDV, rice black streaked dwarf virus; RDV, rice dwarf virus; ROS, reactive oxygen species; RRSV, rice ragged stunt virus; RSV, rice stripe virus; SCF, Skp–Cullin–F-box; SRBSDV, southern rice black-streaked dwarf virus; TCP, TEOSINTE BRANCHED/CYCLOIDEA/PCF; TIRI, transport inhibitor response protein 1; TMV, tobacco mosaic virus.

JA is a key regulator of plant development, as well as defense reactions to necrotrophic pathogens and insect infestation [8]. Unlike SAR induced by SA, JA together with ET generally regulates induced systemic resistance (ISR), another important type of systemic defense response [7]. Although an increasing number of reports highlight the importance of JA in plant–virus interactions [3,5,26,27], the virus–JA relationship is ambiguous. Several studies have outlined the positive roles of JA during compatible interactions [6,28]; for example, continuous JA treatment decreased the DNA titer of beet curly top virus (BCTV), indicating that suppression of the JA response may be critical for geminivirus infection [28]. Cucumber mosaic virus (CMV) 2b protein targets JA signaling in *Arabidopsis* by directly interacting with JA ZIM domain (JAZ) proteins, thus increasing the host attractiveness to insect vectors for virus transmission [26]. However, JA signaling does not always protect against viral infection: Oka and colleagues (2013) reported that the silencing of genes encoding a JA biosynthetic enzyme or the JA receptor CORONATINE INSENSITIVE 1 (COI1) reduced viral accumulation and pathogenesis [29]. Moreover, in transgenic plants expressing viral suppressors of RNAi (VSR) proteins, the expression of JA-responsive genes was repressed. These VSRs included HC-Pro encoded by potato virus Y (PVY), p25 encoded by potato virus X (PVX), 126 KDa replication protein encoded by tobacco mosaic virus (TMV), and 2b encoded by the subgroup II CMV strain KIN, as well as geminivirus C2 and βC1 [30]. Recently, other studies have also associated JA with the RNA silencing pathway. Suppression of *TEOSINTE BRANCHED/CYCLOIDEA/PCF* (*TCP*) expression and JA-mediated plant resistance are observed during viral infection via microRNAs [31]. In addition, a more comprehensive study with a tenuivirus RSV demonstrated that coat protein (CP) of RSV triggers JA biosynthesis and signaling and further up-regulates JA-induced MYB (JAMYB) transcription factor (TF) to initiate the ARGONAUTE 18 (AGO18)-mediated host defense network [27,32] (Fig 3A). But how RSV CP protein induces JA synthesis remains to be elucidated. Nevertheless, these studies allure more attention to phytohormones and RNA silencing crosstalking.

### The impact of SA–JA crosstalk

In addition to the individual functions of SA and JA, their crosstalk is important in regulating the signaling pathways involved in plant–virus interactions [6,7,33]. Several components mediate the antagonistic interaction between SA and JA, including NONEXPRESSOR OF PATHOGENESIS-RELATED GENES 1 (NPR1) and several TFs [34]. The cauliflower mosaic virus (CaMV) P6 protein suppressed SA signaling by interacting with cytosolic NPR1 and affecting its localization, leading to a modulation of JA response in P6 transgenic plants [35,36]. In addition, the CP of *alfalfa mosaic virus* (AMV) interacted with and inhibited the function of ILR3, a member of the basic helix–loop–helix (bHLH) TF family. Infection of *ilr3*.*2* plants with AMV increased the level of JA but reduced that of SA, indicating the presence of antagonist crosstalk [37]. Antagonism between JA and SA also disrupts hormone synthesis or signaling. For example, Oka and colleagues (2013) demonstrated that silencing the JA biosynthesis enzyme ALLENE OXIDE SYNTHASE (AOS) or the JA receptor COI1 enhanced plant resistance to TMV and led to an increased SA level in *COI1-* or *AOS*-silenced plants, which reduced the accumulation of TMV in tobacco containing the *N* gene. These results indicate the importance of the endogenous JA and SA levels in determining the degree of resistance [29].

JA and Methyl Jasmonate (MeJA) initially accumulate in the phloem exudates of leaves upon TMV infection, which is followed by the accumulation of SA. Notably, plants that are impaired in the JA pathway fail to accumulate SA, indicating that JA or MeJA is required for SA accumulation. The JA- and/or MeJA-required SA accumulation suggests a mechanism for the interaction between these 2 hormones in the systemic resistance response against TMV [6]. In another example, the SA treatment reduced necrosis induced by a synergistic infection of both PVX and PVY. It was further shown that the reduced necrosis was dependent on an antagonistic relationship between JA and SA signaling pathways [38]. Crosstalk between JA and SA pathways has also been demonstrated in the regulation of defense gene expression. It was recently shown that application of SA or JA induced the expression of the mitogen-activated protein kinase (MAPK3) defense gene and conferred resistance to tomato yellow leaf curl virus (TYLCV), suggesting important roles of SA and JA in the MAPK3 antiviral pathway [39].

### ABA/ET and plant–virus interactions

The phytohormone ABA functions in diverse plant processes, including the regulation of stomatal aperture and the initiation of adaptive responses to various biotic and abiotic stresses [40]. Although the involvement of ABA in plant pathogen responses has been studied extensively, its role in virus infection is not well characterized [5,41]. Knowledge concerning ABA functions within the complex interactions among virus infection and host–plant defense mechanisms is limited. One study showed that ABA can negatively regulate rice defense against rice black streaked dwarf virus (RBSDV) infection via suppression of JA pathway and ROS accumulation in plants [42]. Several bioassay-based reports focus on 2 of the molecular mechanisms that underlie ABA-mediated plant–virus interactions: callose deposition at plasmodesmata (PD) [43,44] and the RNAi pathway [11].

### ABA-mediated effects

The induction of hormones by virus infection is a common phenomenon during plant–virus interactions; however, the reported effects of viral infection on ABA concentration vary. An increase in ABA content following virus infection has been reported in a number of virus–host interaction systems [41,45]. For example, tomato plants harboring the *Tm-1* gene for resistance to TMV contained elevated concentrations of ABA compared with susceptible plants [45] and infection of *Nicotiana benthamiana* with bamboo mosaic virus (BaMV) or CMV increased the ABA content [41], whereas Rajagopal (1977) demonstrated a decrease in ABA concentration in TMV-infected tobacco at early stages of virus infection [46]. In other studies, virus infection did not induce ABA, such as during the early response of potato to potato virus Y^NTN^ (PVY^NTN^) infection [47]. Exogenous application of ABA increased virus resistance [41,48,49], indicating an antiviral role for ABA. This was also demonstrated by experiments in which ABA biosynthesis was inhibited and/or that used ABA-deficient mutants [41,49,50]. A recent study showed that mutations in *ABA-DEFICIENT 1* (*aba1*), *ABA-DEFICIENT 2* (*aba2*), *ABA-DEFICIENT 3* (*aba3*), or *ABA-INSENSITIVE 4* (*abi4*) accelerated systemic TMV-cg accumulation in *Arabidopsis* [49]. Another study demonstrated that mutants downstream of the ABA pathway [*abscisic aldehyde oxidase 3* (*aao3*), *abscisic acid insensitive 1–1* (*abi1-1*), *abi3-1*, and *abi4-1*] were susceptible to BaMV [41]. Iriti and colleagues (2008) also demonstrated that exogenous ABA application induced significant resistance to tobacco necrosis virus (TNV), which was reduced in the presence of the ABA inhibitor nordihydroguaiaretic acid (NDGA) [50].

The intercellular spread of plant viruses involves movement of the viral genome or virion through PD. The PD conductivity is regulated by the controlled buildup of callose at the PD neck. This is mostly mediated via the antagonistic action between callose synthases and β-1,3-glucanases [44,51]. The involvement of ABA in primed callose production is one of the few examples reporting a positive role of ABA in virus disease resistance [52,53]. One relevant study showed that ABA inhibited the transcription of a basic β-1,3-glucanase that can degrade β-1,3-glucan callose, thus forming a physical barrier to viral spread through PD [52]. Disrupting the expression of basic β-1,3-glucanase conferred increased resistance to viral infection [54]. In addition, a subset of type 2C protein phosphatase (PP2C) genes was specifically up-regulated in *R* gene–mediated extreme resistance against *soybean mosaic virus* (SMV) [53]. This resistance is due to the SMV CI protein and is accompanied by ABA accumulation and callose deposition. Overexpression of PP2C activated callose deposition and inhibited virus movement, indicating that PP2C negatively regulates virus movement and is a signaling component that links ABA accumulation with callose deposition [53]. Therefore, ABA can function as a resistance factor in plant–virus interactions by modulating callose deposition, which has the potential for further study. It is an interesting question how SMV CI mediates ABA accumulation.

The RNAi pathway is a well-known major defense mechanism against viruses within plants [32], and ABA also modulates plant defenses at the level of the RNAi machinery. Recent reports showed that disturbances in small RNA pathways by loss of function of all 4 DCLs enhanced ABA response in *Arabidopsis* [55], indicating that the biogenesis of 1 or more specific microRNAs functions in ABA signaling. The close relationship between ABA signaling and the RNAi pathway was also demonstrated by ABA mutants (*aba1-5*), in which the expression level of AGO1 was significantly increased, suggesting that ABA regulates the AGO1 protein [56]. The same study also showed that *MIR168a*, a negative regulator of AGO1 that is often induced during virus infection, contains ABA-responsive elements within its promoter [56,57]. A strong link between ABA-mediated signaling and RNA silencing was recently demonstrated. AGO3 plays an important role in RNA silencing and functions upon infection of *N*. *benthamiana* with potato spindle tuber viroid (PSTVd), and ABA exposure leads to AGO3-mediated resistance to BaMV infection [12,58]. In view of the regulatory role of ABA in the RNAi pathway, these functions in virus infection need to be further characterized.

### ET-mediated effects

ET is the simplest unsaturated hydrocarbon gas molecule and functions in many plant developmental stages, such as senescence and in defense responses against necrotrophic pathogens [9,19]. ET is induced upon virus infection and is associated with symptom development [59,60]. The role of ET in plant defense against viruses is somewhat controversial; it contributes to resistance in some interactions but promotes disease progression in others [1,9,10,49,61–64]. Viruses also use ET signaling to invade their hosts, by producing ET-inducing effectors that converges on ET signaling to disrupt plant immunity [65–68].

Several groups have demonstrated that ET participates in the development of viral symptoms. Using a temperature-induced synchronous lesion formation system, Ohtsubo and colleagues (1999) showed that increased ET production preceded and promoted lesion formation. In addition, inhibitors of ET biosynthesis or action significantly suppressed lesion formation [59]. Another piece of evidence that links ET to viral symptom development is the function of the CaMV P6 protein, which is involved in virus replication and RNAi suppression. *Arabidopsis* mutants that suppress the phenotype induced by the transgenic expression of CaMV P6 are less susceptible to CaMV infection and show reduced ET sensitivity [60]. The authors concluded that P6 interacts with components of the ET signaling pathway and that P6 transgenic plants are more resistant to CaMV infection. However, some reports claimed that transgenic tobacco plants with altered ET levels or reduced sensitivity to ET were not impaired in their ability to form local lesions after TMV infection [69,70], and the HR response of *Arabidopsis* to turnip crinkle virus (TCV) was not dependent on ET [71]. Therefore, the role of ET in the development of necrotic lesions remains contentious and requires clarification.

Overwhelming evidence suggests that the ET pathway play a bidirectional role in plant–virus interactions. An experiment using the ET signaling pathway mutants *ethylene insensitive 2* (*ein2*) and *ethylene response 1* (*etr1*) showed an enhanced resistance to CaMV infection [62]. Other mutants in the ET pathway, such as *1-aminocyclopropane-1-carboxylate synthase* (*acs1*), *ethylene-responsive transcription factor 106* (*erf106*), and *ein2*, are also resistant to TMV-cg. The ET signaling pathway was also correlated with suppression of the defense response to turnip mosaic virus (TuMV) and enhanced reproduction of the green aphid, *Myzus persicae* in *Arabidopsis* [10]. Transgenic expression of Nuclear Inclusion a-Protease (NIa-Pro) from TuMV alters ET responses and suppresses aphid-induced callose defenses. Consistent with these results, a recent report outlines a mechanism by which the rice dwarf virus (RDV) hijacks host factors via enhancing the enzymatic activity of S-adenosyl-l-methionine synthase 1 (SAMS1) and increasing ET production or signaling, thereby reducing the host antiviral defense response and enhancing virus infection and accumulation [9]. However, there are 2 reports showed controversial results. Application of the ET precursor 1-aminocyclopropane-1-carboxylic acid (ACC) promoted the accumulation of TMV-cg in systemaic leaves [49]. Chilli veinal mottle virus (ChiVMV) regulates expression of *N* gene, while JA and ET signaling is essential for systemic resistance to ChiVMV in tobacco [64]. The reason for the contrary roles of ET may depend on different virus–host combination and the crosstalk among ET and other phytohormones. It would be worthwhile to distinguish ET synthesis and signaling pathways in these research and, in particular, to include genetic data.

During crosstalk mediated by different phytohormones, ET perception is also required to establish SAR in TMV-infected leaves, which eventually triggers SA accumulation and SAR development in uninfected leaves [66]. Treatment of tomato plants with *Trichoderma harzianum* strain T-22 (T22) led to systemic resistance against CMV infection through JA/ET and SA signaling pathways [72]. Alternatively, overexpression of *NtERF5*, an ET-responsive TF from *Nicotiana tabacum*, caused enhanced resistance of transgenic tobacco plants to TMV [67]. The ET pathway is also correlated with reactive ROS that was induced systemically after CaMV infection, and the ROS accumulation was dependent on ET and NADPH oxidase [65,68]. Clearly, multiple defense pathways operate through ET pathways in response to viral infection.

### The impact of ABA–ET crosstalk

Studies on crosstalk have focused on the interaction between sugar signaling mediated by ABA and ET, whereby high ABA concentrations inhibit ET production [73,74]. Although data are rapidly accumulating in support of the antagonistic interaction between ABA and ET in plant development, little is known about the interactive function of ABA/ET in plant–virus interactions. Studies have demonstrated increased levels of ABA and ET in CMV-infected cucumbers, and both hormones are involved in suppressing hypocotyl elongation [75,76]. Chen and colleagues (2013) reported that the WRKY8 TF inhibited TMV-cg infection by regulating ABA and ET pathways, indicating crosstalk between ABA and ET signaling during TMV-cg–*Arabidopsis* interaction [49]. These reports demonstrate that ABA–ET interactions might differentially affect plant–virus homeostasis. Unfortunately, in-depth studies on this topic are lacking. It remains to be determined how ET regulates host response to virus infection in these cases.

### Auxin/GA and plant–virus interactions

#### Auxin-mediated effects

Although it is difficult to formulate general rules about the effects of viruses on phytohormones, viruses clearly affect auxin and GA individually or combinatorially, depending on the virus–host combination. Auxin is an important phytohormone with roles in plant growth and development [77], but a number of reports also demonstrated that auxin signaling pathway plays an important role in plant–virus interactions (Fig 3B). Viruses can hijack the auxin signaling pathway by disrupting the localization or function of the Aux/AR repressor proteins. For example, the TMV replication protein interacted with and disrupted the nuclear localization of Aux/IAA proteins thereby affected auxin-mediated gene regulation and disease development (e.g., by enhancing TMV phloem loading) [13,78,79]. A recent study showed that the P2 capsid protein from RDV physically interacts with OsIAA10 and prevents its degradation. Consequently, RDV-infected plants are dwarf and display excessive tillering [14]. A more comprehensive study showed that OsIAA10 interacted with specific auxin response factors (ARFs) in plants, and antiviral functions of these OsARFs are diversified. We hypothesized that there may be several pathways between rice resistance to RDV infections. First, the auxin-IAA10-dependent pathway based on the interactions between IAA10 and ARF family members, such as IAA10 interaction with OsARF11, OsARF12, and OsARF16. Among those ARFs, OsARF12 and OsARF16 positively regulate rice defense against RDV infection, while OsARF11 negatively regulates rice resistance to RDV infection. Second, there is an auxin-dependent but IAA10-independent pathway. For example, OsARF5 does not interact with IAA10 but still negatively regulates rice resistance to RDV infection. The existence of this auxin-dependent but IAA10-independent pathway suggests that other OsIAAs or other OsIAA-independent pathways might participate in rice antiviral defense against RDV infection [80].

Many viral infections result in aberrant phenotypes that parallel the involvement of auxin in virus establishment. Vitti and colleagues (2013) reported that root growth was accompanied by significant changes in the levels of IAA proteins during CMV infection [81]. ARFs are also key viral targets in host–virus interactions. Three different viral silencing suppressors cause developmental abnormalities by misregulating a miR167-targeted ARF [82], and 2 fijiviruses and a tenuivirus also target OsARF17 to perturb auxin signaling and facilitate infection [83]. These and other results not mentioned here pinpoint to the complexity of auxin signaling during plant–virus interactions.

#### GA-mediated effects

In addition to disrupting auxin signaling, virus infection also affects GA pathways. GAs are diterpenoid hormones that control various aspects of plant physiological development [84,85], but little is known about their function in plant–virus systems. Extensive studies have focused on rice–virus interactions. Zhu and colleagues (2005) observed that RDV infection deceased the levels of GAs, and symptom development could be alleviated by GA application. Further study revealed that the RDV-encoded P2 protein interacted with *ent*-kaurene oxidases, which catalyze 1 step in GA biosynthesis [15]. It should be kept in mind that RDV P2 also interacts with *ent-kaurene* oxidase-like (KOL) proteins, which have a high similarity in amino acid sequence with *ent-kaurene* oxidase, and these KOL proteins may be involved in phytoalexin biosynthesis [86]. In addition, a P7-2 protein encoded by RBSDV, which is a devastating viral pathogen that causes severe symptoms in infected plants, was confirmed to interact with GIBBERELLIN-INSENSITIVE DWARF2 (GID2) in rice and maize. GID2 is an essential regulator of the GA signaling pathway [18]. RBSDV invasion reduces active GAs accumulation [87]. These results indicate an important role of GAs during RBSDV infection. The GA pathway might also function in plant–virus interactions together with small RNAs. Recently, a study showed that increased levels of miR167 resulted in a decreased concentration of GA in PVY-infected potato [88]. This revealed a previously undescribed connection between small RNAs and GA biosynthesis, thereby representing an important link between defense and developmental signaling pathways.

#### The impact of auxin–GA crosstalk

The functions of auxin and GAs overlap in the regulation of multiple aspects of plant development, most of which relate to cell expansion and differentiation. For example, the level of auxin due to its biosynthesis and transport controls DELLA protein abundance, and DELLA proteins also integrate signals from ET, auxin, and GAs during growth [89,90]. Although the interactions between GAs and auxin have been intensively investigated, those between GAs and auxin signaling in plant immunity have not been analyzed in detail. The application of exogenous auxin repressed the virus-inducible dwarf 1 (*vid1*) phenotype of turnip vein clearing virus (TVCV), whereas the application of GA had no effect [91]. Zhu and colleagues (2005) also showed that treatment of RDV-infected plants with exogenous GA restored the non-dwarf phenotype, but treatments with auxin did not [15]. Although GA treatment restores the defect of growth and development caused by RDV infection, the virus accumulation level is not reduced significantly. Therefore, the interaction between GAs and auxin upon viral infection is unclear, and a potential integrated response involving both 2 hormones may exist. Whether GA and the GA signaling has the potential antiviral function should be investigated, in light of their functions in plant growth regulation in infected plants.

### CKs and plant–virus interactions

CKs act as long-range and local signals and are involved in multiple regulatory processes throughout plant development [92]. CK signaling is also important for positively or negatively modulating plant innate immunity [19]. Several pathogens also produce CKs, which may be essential for their infectivity [93,94]. Several early investigations attempted to quantify the changes in endogenous CKs that occur following plant infection by viruses [95,96]. The greatest changes in endogenous CKs occurred in roots 4 days after primary PVY^NTN^ infection in soil-grown potato plants, when a shift in the concentration of biologically active free CKs toward inactive 9-glucosides was observed [97]. In addition, Clarke and colleagues (1999) demonstrated a specific decrease in the level of active CKs in the first few days following infection by *white clover mosaic virus* (WClMV) and showed that production of the inactive 9-glucoside form was a direct response to WClMV infection [98]. Only few reports describe the response of CK pathways to virus infection; for example, the geminivirus AC2/AL2 protein interacted with an adenosine kinase in *Arabidopsis* and led to the increased expression of primary CK-responsive genes [16]. Further study is required to establish which CKs-mediated crosstalk is involved in plant–virus interactions.

### BRs and plant–virus interactions

BRs are steroid plant hormones that have been extensively studied since their initial isolation and characterization [99]. They regulate various aspects of plant growth and development [100], but also function in plant immunity by inducing plant defenses against viruses [101]. The levels of castasterone and deoxocastasterone, which are the important BR biosynthesis intermediates, increased in TMV-infected tobacco leaves. Treatment of tobacco plants with brassinolide (BL) led to enhanced resistance against TMV but not to SA accumulation or the induction of *PR* gene expression, indicating that BL-induced resistance is distinct from SAR [101]. Reports also showed that BR signaling was critical for the induction of tolerance against CMV [17]. This is extremely significant for understanding the relationship between BR-mediated signaling and genes such as *WRKY30* in response to viruses. Components of BR pathways also play important roles during plant–virus interactions. For example, BRASSINOSTEROID-INSENSITIVE 2 (BIN2), a negative regulator of BR signaling, interacted with the geminivirus C4 protein [102], although the significance of this interaction remains unclear. Another component, BRI1-associated receptor kinase 1 (BAK1), which is a co-receptor in BR signaling, was identified to play important functions in some RNA virus infections; for example, TCV, oilseed rape mosaic virus (ORMV), and TMV accumulated to higher levels in *bak1-4* and *bak1-5* mutants than in wild-type plants [103]. Advances in the area of BR-mediated plant–virus interactions require further research.

### Other hormones crosstalking in plant–virus interactions

Interactions between hosts and their corresponding viruses are extensive and overlapping. To elucidate these affects, the crosstalk among different phytohormones that enables plants to fine-tune energy allocation and minimize fitness costs through flexible complex signaling networks should be characterized. Although antagonistic crosstalk between SA and JA/ET signaling pathways is well known, SA and ET also function synergistically to induce resistance against CMV in *Arabidopsis* [104]. Fine mapping and sequence comparison identified a *RCY1* gene, which encodes 104-kDa CC-NBS-LRR type protein. The *RCY1*-conferred resistance requires both SA and ET signaling but not JA signaling [104,105]. Crosstalk also exists between SA and ABA; for example, BaMV and CMV infection activates SA and ABA pathways in *N*. *benthamiana* [41]. Furthermore, SA and ABA homeostasis was involved in the resistance of tomato and *Arabidopsis* to TYLCV [106]. The TMV-cg CP protein was also demonstrated to stabilize DELLA proteins, thereby modulating GA- and SA-mediated defense pathways [107]. The WRKY8 TF induced a gene related to ABA signaling, but repressed genes involved in ET signaling by binding to the promoters of genes involved in both pathways. Moreover, WRKY TFs mediate the long-distance movement of viruses and crosstalk between the ABA and ET signaling pathways to modulate antiviral resistance mechanisms [49]. Very recently, JA and BR crosstalk has been found to play an important role in response to RSV infection. The authors found that RSV infection significantly inhibits the BR signaling pathway and increases the accumulation of OsGSK2, which interacts with and phosphorylates OsMYC2, resulting in the degradation of OsMYC2 and reducing JA-mediated RSV resistance [108]. Another study also showed that JA-mediated defense could suppress the BR-mediated susceptibility of rice to RBSDV infection [109]. Infection by RBSDV might also influence the GA pathway [18]; thus, RBSDV may interfere with different phytohormone crosstalk pathways to counteract plant immune responses, which should be investigated further. Because GAs are biosynthesized from common intermediates shared by both CK and ABA, potential integrated responses that involve all 3 hormones should warrant further study.

## Conclusions and perspectives

The disruption of phytohormone biosynthesis and signaling pathways following virus infection is extremely complex. Significant progress has been made in elucidating the molecular mechanisms that underlie crosstalk among different phytohormones during virus infection. Changes in endogenous phytohormone levels appear to be a direct consequence of virus infection and are tightly coordinated with viral movement, replication, symptom development, and defense responses. Table 1 summarizes the commonly used marker genes in response to phytohormones and the analysis methods for different phytohormones during plant–virus interactions. Hijacking host components in the phytohormones pathways is also a common strategy in viral pathogenesis. The evolution of interactions between phytohormone signaling networks and viral infection is a recurring theme. Current studies are extending knowledge concerning the host manipulation hypothesis, as well as the alterations, conservation, and diversification within phytohormone signaling networks during viral infection. The challenge in forthcoming years lies in identifying the roles of phytohormones in plant–virus interactions and crosstalk among different hormone pathways and in translating the basic knowledge gained from model species to crops.

**Table 1:** Summary of marker genes and quantitive methods of phytohormones in plant–virus interactions.

Phytohormones	Responsive marker genes^a^	Quantitive methods	Reference(s)
SA	*PR1*, *PR2*, *PR5*	GC^**b**^–MS^**c**^, LC^**d**^–MS	[6,88]
JA	*PDF1*.*2*, *PR3*	GC–MS, LC–MS	[6,88,108]
ET	*ERF1*, *ETR1*	GC	[9,10]
GAs	*GAMYB*	GC–MS, LC–MS, ELISA	[15,85,87,88]
ABA	*ABF1/2/3/4*	UPLC^**e**^-MS/MS, GC–MS, ELISA	[41,42,55,56,88]
Auxin	*IAA1/5/19*, *GH3*.*3*	GC–MS, LC–MS	[14,81,88]
CKs	*Type-A ARRs*	LC–MS	[16,81,87,92,93]
BRs	*BES1/BZR1*	GC–MS, LC–MS/MS	[101,108]

a These genes are *Arabidopsis* or rice-derived, plant species and organ should be taken into consideration in marker gene selection.

b GC, gas chromatography.

c MS, mass spectrometry.

d LC, high performance liquid chromatography.

e UPLC, ultra performance liquid chromatography.

ABA, abscisic acid; BR, brassinosteroid; CK, cytokinin; ET, ethylene; GA, gibberellic acid; JA, jasmonic acid; SA, salicylic acid.
